# On the Robustness of In- and Out-Components in a Temporal Network

**DOI:** 10.1371/journal.pone.0055223

**Published:** 2013-02-06

**Authors:** Mario Konschake, Hartmut H. K. Lentz, Franz J. Conraths, Philipp Hövel, Thomas Selhorst

**Affiliations:** 1 Institut für Epidemiologie, Friedrich-Loeffler-Institut, Wusterhausen, Germany; 2 Institut für Theoretische Physik, Technische Universität Berlin, Berlin, Germany; 3 Institut für Physik, Humboldt-Universität zu Berlin, Berlin, Germany; 4 Bernstein Center for Computational Neuroscience, Humboldt-Universität zu Berlin, Berlin, Germany; 5 Center for Complex Network Research, Northeastern University, Boston, Massachusetts, United States of America; INSERM & Universite Pierre et Marie Curie, France

## Abstract

**Background:**

Many networks exhibit time-dependent topologies, where an edge only exists during a certain period of time. The first measurements of such networks are very recent so that a profound theoretical understanding is still lacking. In this work, we focus on the propagation properties of infectious diseases in time-dependent networks. In particular, we analyze a dataset containing livestock trade movements. The corresponding networks are known to be a major route for the spread of animal diseases. In this context chronology is crucial. A disease can only spread if the temporal sequence of trade contacts forms a chain of causality. Therefore, the identification of relevant nodes under time-varying network topologies is of great interest for the implementation of counteractions.

**Methodology/Findings:**

We find that a time-aggregated approach might fail to identify epidemiologically relevant nodes. Hence, we explore the adaptability of the concept of centrality of nodes to temporal networks using a data-driven approach on the example of animal trade. We utilize the size of the in- and out-component of nodes as centrality measures. Both measures are refined to gain full awareness of the time-dependent topology and finite infectious periods. We show that the size of the components exhibit strong temporal heterogeneities. In particular, we find that the size of the components is overestimated in time-aggregated networks. For disease control, however, a risk assessment independent of time and specific disease properties is usually favored. We therefore explore the disease parameter range, in which a time-independent identification of central nodes remains possible.

**Conclusions:**

We find a ranking of nodes according to their component sizes reasonably stable for a wide range of infectious periods. Samples based on this ranking are robust enough against varying disease parameters and hence are promising tools for disease control.

## Introduction

Animal trade represents an important economic sector. At the same time, it also provides a major route for economically most important infectious livestock diseases [1]–[4] as has been shown for foot-and-mouth disease [5], [6] and classical swine fever [7]. Any efficient disease mitigation or prevention strategy therefore needs to include animal trade in its considerations. However, control measures themselves may cause tremendous animal welfare and economical problems within the agricultural production chain. A careful assessment of risk is important and should be one of the main goals of modern epidemiology.

Epidemiology has been influenced by network science in recent years [8]. Animal trade can be described as a network by representing the agricultural holdings by nodes, which are connected to each other by directed edges. Traditionally, time-aggregated static networks were studied, where an edge 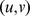 exists when at least one contact between the nodes 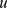 and 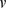 is recorded during the period of observation, as reviewed by Martínez-López et al. [9] and Dubé et al. [6]. Recent research, has shown, however, that this static network representation of animal trade is inappropriate for epidemiological purposes [10]–[15]. Vernon et al. [11] point out that the spread of infectious diseases is only predicted correctly if the chronology of contacts is accurately reflected.

One way to meet this demand is the utilization of time-dependent networks, also known as temporal networks. In temporal networks an edge is represented by a triple 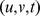, where 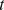 is its time of occurrence. For animal trade networks the contacts may be assumed as instantaneous due to the short transportation time compared to the length of stay in a holding. This is reflected by the temporal resolution of the available trade data as described in the Materials section. Such time-discrete temporal networks can be visualized as a stack of graphlets [16], where a graphlet is a static snapshot of the trade at any discrete moment 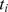. The temporal network itself is then represented by all graphlets stacked on top of each other in the correct order.

In their recent review on temporal networks, Holme and Saramäki [17] point out that a formalism to treat these objects is still lacking. Instead there are many parallel developments made in various disciplines ranging from biology to computer science and sociology. Nevertheless Danon et al. [8] call the development of such a formalism one of the most pressing issues of network epidemiology. However. only very recent contributions have been made in this direction [18], [19].

In fact, there has already been research on temporal networks in the context of epidemic spreading. Vernon et al. [11] modeled an SIR-(susceptible-infectious-recovered) and Natale et al. [20] an SI-like disease on the British and Italian cattle trade networks, respectively. Other works used mobile phone or email datasets to study disease dynamics on temporal networks. Furthermore, Vazquez et al. [21] and Karsai et al. [22] modeled an SI-like spreading process and found that it is slowed down due to the temporal structure of the data. Interestingly, Rocha et al. [23] observed the opposite behavior in a dataset of sexual contacts in Internet-mediated prostitution. Similarly, Miritello et al. [24] found an SIR-like disease on a mobile phone call dataset to spread more efficiently for small values of the transmission probabilities, but less efficiently for higher values. Stéhle et al. [25] studied a face-to-face contact network of conference attendees and find it well approximated by a weighted time-aggregated network concerning the course of an SEIR-modeled disease, which takes into account an additional intermediate *exposed* state. Moreover, a basic model to study the effect of the distribution of inter-event times has been recently proposed by Rocha et al. [26].

Together with a better understanding of the initial spread of a disease [2] and the identification of influential spreaders [27], the development of disease control strategies is of most importance in this area. Lee et al. [28] propose a vaccination strategy for temporal networks, which is an adaptation of the well-known neighborhood vaccination protocol [29] and hence only relies on local information. In cases, where global information about the network topology is available, the identification of risk-based central nodes seems to be a more promising strategy. For animal trade networks, this spatial and temporal information is usually available due to legal obligations [30].

The term risk-based centrality is context-dependent and has at least a two-fold meaning. It can either characterize the potential of one node to infect other nodes or it can characterize the exposure of a node of being infected by others. Many of the well-established centrality measures for static networks have already been adapted for temporal networks [17], [31]–[37], but none of them explicitly relates the timescale of the dynamic process on the node level, i.e. the duration of the local infection dynamics. However, most epidemiological models crucially depend upon finite and fixed infection timescales representing an infectious period. To our knowledge, the only contribution proposing a measure including variable infectious periods was made by Natale et al. [12], where a disease flow centrality is presented as a measure tailor-made for animal trade networks.

The difficulty to define a risk-based centrality is caused by the complexity of any centrality measure that considers finite dynamic timescales. Even worse, the centrality of a node will not only depend on the infectious period 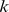 [25], but also on the time of infection 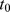 of the specific node itself. A particular node might be central if it is infected at one particular moment, but might drop below average after a short period of time. A chain of causation must therefore be preserved in the temporal network.

To our knowledge, these dependencies have so far not been investigated systematically. This paper attempts to start filling this gap. To this end, we investigate the temporal robustness of two simple measures of centrality for an SIR-like disease spread on the German pig trade network.

Temporal robustness means that a centrality measure is insensitive to variations in the time of infection 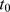 and the infectious period 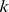. The intuitive understanding of centrality and risk is rather time-independent, i.e. it seems to be suitable to assign a time-independent value of centrality to a node than assigning a function of 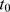 and 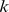.

Here, we try to answer the question to what extent this is still feasible in the context of temporal networks. Particularly we focus on the case of epidemiological relevant centrality in the context of network topologies significantly changing on the timescale of a typical infectious period.

A frequently used measure in epidemiology is the final size of an epidemic, which is the number of all infected individuals throughout an epidemic. In network terminology, this is equivalent to the number of nodes that can be reached from a primarily infected node, i.e. the size of its out-component, when a transmission probability of 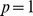 is assumed. The number of nodes that can be reached from a particular node, defines a measure of its centrality [38], which is also known as *virulence*. A similar concept called *reachability* was discussed in [39] for communication networks, where a time-ordered list of contacts was taken into account.

Another measure of centrality is defined by its reversal, i.e. the number of nodes from which a particular node can be reached. This number is given by the size of its in-component and corresponds to a *vulnerability* of the node. The epidemiological importance of this feature has already been emphasized by Riolo et al. [40]. In a recent work, Kivelä et al. analyzed the spreading in a large-scale communication network based on mobile-phone calls, where – similar to the current study – a transmission probability 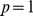 was assumed [41]. They found an upper bound for the speed of spreading mediated by the network and compared it to randomized reference models, which preserved selected correlations.

Node components have already been used for risk assessments in static representations of animal trade networks [42]–[44]. Since both measures can be intuitively extended to non-static topologies, Nöremark et al. [13] and Dubé et al. [10], for instance, introduced the out- and ingoing infection chain as a risk-based measure that respects the temporal sequence of contacts. They did not consider finite infectious periods, i.e. dynamic timescales in nodes. However, the very concept of either measure can be intuitively extended to take also finite infectious periods into account.

We make use of the out-component 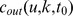 and the in-component 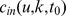 of a node 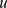 in a way that respects the temporal sequence of contacts, finite infectious periods 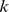 as well as their time of infection 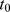. From an epidemiological point of view, both measures 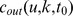 and 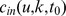 are relevant. The size of the out-component of a node gives an upper bound of the size of any epidemic starting in this very node, while the size of the in-component of a node is proportional to the probability of getting infected if an epidemic starts somewhere in the network. For the sake of simplicity, counteractions and network adoption to the epidemic are neglected, i.e. we study the effects of undetected spreading of a disease under normal trading conditions. We will demonstrate how the time-dependent approach is superior for a risk assessment in terms of the the maximum number of potentially infected nodes.

In the following, we first present the data, on which the analysis is based, and then the algorithm used to calculate out- and in-components. We then investigate the dependence of both measures on the time of infection 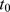 and the length of the infectious period 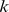. We compare the results with the time-aggregated network as a reference. Other models involving for instance temporal shuffling [25], [41] of edges, represent also possible reference cases, but are beyond the scope of this present paper.

We propose conditions under which centrality can be assigned independently of 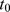 and 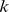. We find that in spite of strong temporal heterogeneities, samples based on a ranking due to the size of out- and in-component are robust enough for practical concerns of disease control.

## Materials and Methods

The data used in this paper is an excerpt of HIT [45], the national German database on pig trade established according to EU legislation [30]. Whenever live animals are traded, the purchasing and the selling agricultural holding as well as the date of the trade are stored. This data can be interpreted as a temporal network 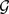, where any such trade contact from holding 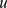 to holding 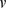 at day 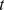 is represented by a directed edge 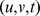. The temporal network 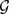 can be interpreted as a time-ordered sequence of static networks 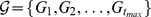, each representing the trade of a single day. The observation period for this paper spans the scope of the years 2008 and 2009 with a total of 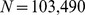 nodes and on average 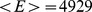 edges per day.

Although the trade of live pigs is subject to seasonal variation and temporal irregularities, we found a period of one year sufficient to obtain a representative picture of the dynamic patterns of the network (see *Supporting Information S1 (Figure S1)*). A simple explanation can be given by the average lifetime of pigs of approximately 180 days, which would let one expect a periodicity of the network of the same order.

In contrast to the temporal network, the static time-aggregated network, where 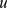 and 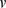 are connected by a directed edge 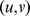 when at least one trade contact between them has been recorded during the observation period, contains 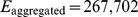 edges. This time-aggregated network exhibits a heavy-tailed degree distribution in both the in- and out-degree, spanning three orders of magnitude. Approximately one third of the nodes belong to a giant strongly-connected component, in which every node is connected to any other node of the component by at least one path. The out-component of these nodes is composed of the giant strongly-connected component itself and all additional nodes that can be reached from it [46]. In our system the size of this out-component is approximately 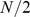.

All other nodes, which cannot reach the giant strongly-connected component, have an out-component with a size three orders of magnitude smaller.

We are not aware of an efficient algorithm to determine the out-component 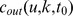 and the in-component 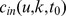 in a temporal network with finite infectious periods 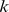. With the introduction of finite infectious periods, the precise time of the primary infection of a node 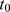 becomes more important and has to be explicitly taken into account. Furthermore it has to be defined, whether multiple visits to the same node are allowed, i.e. if SIS- or SIR-like spreading is assumed. In this paper, we consider a deterministic SIR-like model, where a susceptible holding 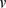 becomes infected with probability 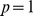, if it has a trade contact 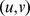 with an infected one 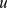. The time of trade is denoted by 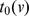. After a time period 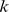, i.e. 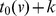, the holding 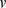 and all its future links are removed from the population. Thus, it does not participate in the spreading process any longer. We assume an SIR-type spreading, because it contains a clear defined breaking condition for the process.

However, the determination of 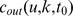 and 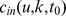 for SIR-type spreading is non-Markovian. The history of a node needs to be considered explicitly, since a node can only become infected if it has never been infected before.

In order to calculate 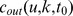 and 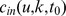, we use a modified breadth-first-search algorithm. We start at a root node 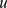 and mark it as infected. At every discrete time step 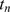, we then identify all edges 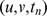 where 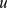 is infected but 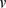 susceptible. All nodes 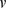 that can be reached this way are marked as infected. Subsequently we iterate over all infected nodes 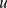 and mark those, for which the infectious period has expired, as *removed*. The infectious period of a node 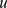 has expired if 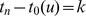, where 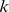 is the infectious period of the disease. Afterwards, the time step 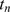 is incremented by one, and we start the next iteration. The search algorithm stops, if no more infected nodes are available.

It is not clear in general, if a given observation period of a temporal network can capture an entire dynamic process on the network [17], [34]. This problem is of minor importance here, as we only consider primary infections 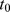 during the first year of the observation period and let the epidemic eventually penetrate into the second year. In this way, we are able to observe any epidemic to vanish and thus, obtain a consistent value for the number of infected nodes.

The nodes visited by our algorithm are identified as the out-component 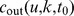 of the root node 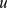, if the temporal sequence of edges in 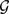 for an initial infection of 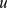 and time 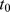 and finite infectious period 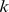 is respected.

The in-component 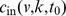 of a node 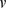 counts the number of times that 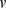 has been visited by the algorithm for a finite infectious period 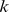 when the algorithm starts at all possible root nodes 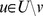 at a time 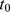. Both components are a function of the time of the primary infection 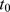 of the root node.

The size of both measures can be conveniently normalized to the number of nodes 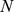. Thereby in- and out-component are bounded in the range 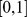.

To investigate their temporal dependency and to gain an understanding of their robustness, we determine both measures for all nodes for infectious periods 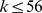 days and for all times 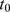. This yields 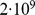 initial conditions for the search algorithm. The choice of 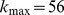 days covers the infectious period of the major livestock diseases [47]. Thus, 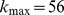 days is assumed to be a reasonable upper value.

## Results

To retain readability, we will restrict the detailed description of results to the analysis of the out-component. The results for the in-component show no conceptual differences and their main figures are replicated in *Supporting Information S2 (Figures S2–S4)*.

### Disease Mitigation and Epidemic Threshold

Before analyzing the robustness of the size of the out-component 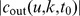, we briefly review the disease mitigating effects of the temporal network structure.

In a time-aggregated representation of a network, any primary infection will cause secondary infections as long as there is at least one outgoing edge during the observation period. In temporal networks, the occurrence of secondary infections is more constrained, as the infectious period 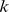 limits the effective time period between the moment of the primary infection 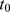 and the occurrence of an outgoing edge.

**Figure 1:**
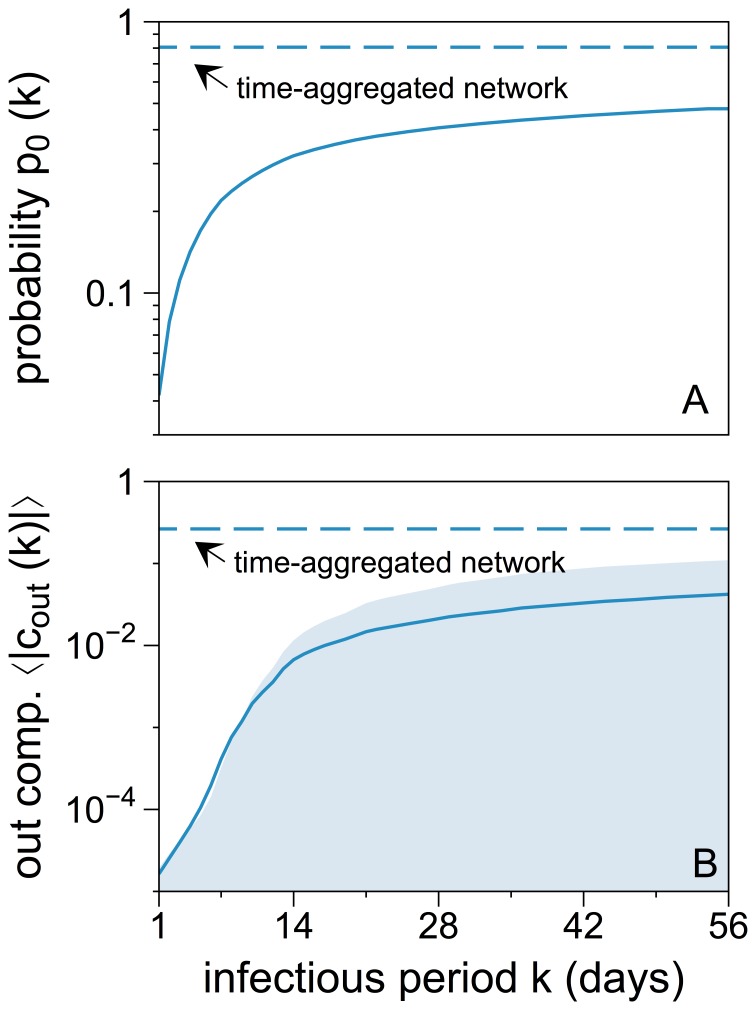
Outbreak probabilities (A) and out-component sizes (B) for different infectious periods 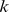. Panel A: Outbreak probability 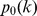 as given by the fraction of primary infections causing at least one secondary infection. The dashed line shows the outbreak probability of the time-aggregated network, i.e. the fraction of nodes with non-vanishing out-degree. Panel B: Average out-components of primary infections, i.e. the *number* of follow-up infections. The 50% confidence interval is indicated by the shaded area. Only for 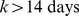 a significant fraction of the network can be infected. For increasing 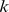, both values approach a saturation. For 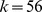 days, approximately every second primary infection will cause follow-up infections which will reach on average 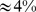 of the network. Both numbers are significantly lower than their counterparts in the static network, as indicated by the dashed line. Here approximately 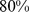 of all primary infections cause follow-up infections with a mean size of epidemic of almost 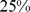 of the network.

Given an infectious period 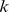, it is possible that 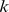 is too small to cause any follow-up outbreak at all. For this reason, we define the *outbreak probability* 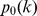 as the fraction of successful secondary outbreaks over the total number of primary infections. Figure 1A shows this outbreak probability as a function of infectious period 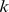 for all simulated primary infections. For small infectious periods, the outbreak probability is close to zero, because causal chains are sparse. For increasing 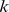, the probability approaches an asymptote, which is defined by the ratio of nodes with at least one outgoing edge in the time-aggregated network. For comparison, this ratio is also plotted as a dashed line. Note that even for 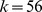 days, the outbreak probability is approximately only half as high as in the time-aggregated case.

Another interesting measure is the average size of the out-component 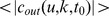 for a given 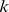, where the average is calculated over all nodes 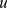 and all times of primary infection 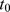. This quantity is shown in Figure 1B. As before, the dashed line refers to the time-aggregated case.

Rocha et al. [23] and Miritello et al. [24] observed a threshold 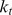 in their publications on SIR-diseases when considering temporal topologies. For 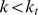 the average size of the epidemic vanishes and increases abruptly at 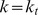. Our results exhibit a similar behavior and show significant values only for 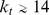 days. As for the outbreak probability, 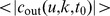 approaches an asymptote that is given by the average size of the out-components in the time-aggregated network. Also for this measure, it should be noted that the average size of the out-component of the time-aggregated network is approximately six times larger, even if 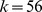 days.

Both observations support our argument that a temporal view on the network is essential to capture its dynamics fully. Calculations based on a time-aggregated network strongly overestimate the size and probability of an outbreak.

### Temporal Heterogeneity of the Out-component

We explored the dependence of the out-component 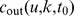 on the time of primary infection 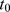 for a node 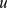.

**Figure 2:**
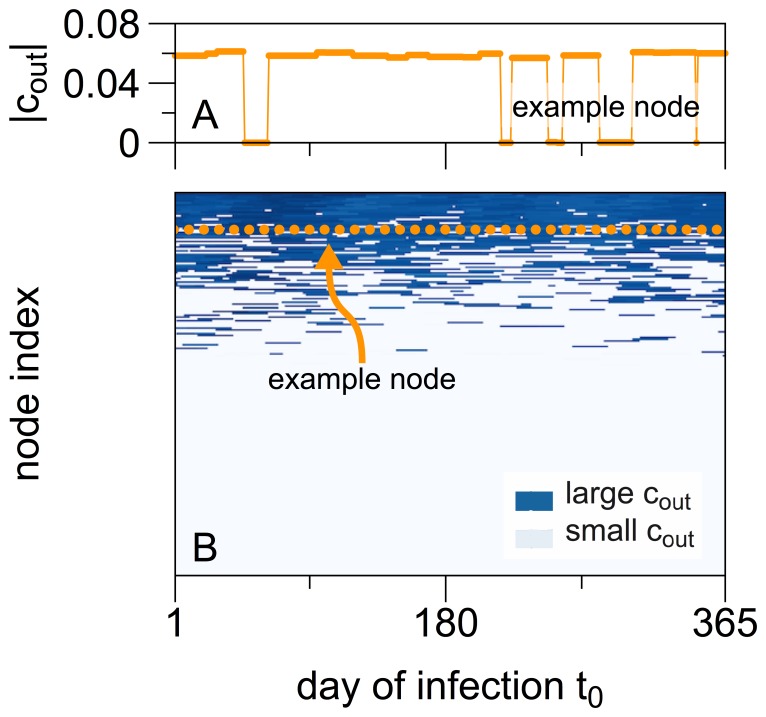
Distribution of 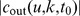 for an exemplary infectious period of 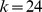 days. Panel A shows the size of the out-component for an exemplary node 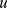 as a function of 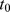. For many times 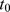, the size of the out-component 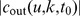 has similar values close to 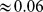, but for some 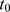 we also find 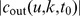 to vanish. Panel B shows the distribution for all nodes, i.e. the top view of panel A for all nodes of the network. Each horizontal line represents one node, the example node chosen for panel A is indicated by the dotted orange line. For the sake of clarity, only every 100th node is plotted. Nodes are arranged according to their averaged value of 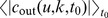 over all 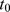 from top to bottom, i.e. the node with the largest averaged out-component is displayed as the top line of the panel.

For illustration purposes we began with an exemplary infectious period of 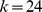 days and the arbitrarily chosen node 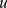. Figure 2A shows the distribution of the size of the out-component 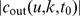. The out-component shows a bimodal behavior. It attained values of 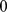 and approximately 6% of the network size. Primary infections with adjacent 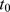 often account for similar out-component sizes.

The explanation lies in the temporal sparsity of edges in the network, as illustrated by the following: Let us assume that a node 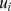 becomes infected at 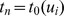 and afterwards connects within its infectious period only to one susceptible node 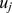 at 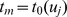, i.e. 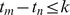. Accordingly, 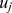 becomes infected. If 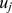 itself has no contacts to other nodes in the time interval 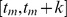, the epidemic stops. Hence, the out-component contains only 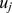. Otherwise, the disease continues to spread and the out-component of 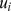 consists of 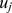 and its out-component. Now, the out-component of 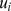 will be the same for all moments of infection 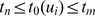. If 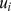 is connected to multiple nodes during its infectious period, small changes in 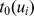 might trigger few more or less infections as a small number of additional nodes enter or leave the causal chain.

Besides small fluctuations of 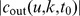, a bi-modality in the distribution was visible. The values of 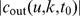 were either close to their maximum or very small. This distribution is related to the existence of a giant strongly-connected component in the time-aggregated network 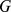. The bi-modally distributed sizes of the out-components of nodes in the time-aggregated network 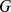 are reflected in the bi-modal distribution of the size of the out-component of a single node at different times of infection 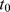 in the temporal network 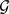. For an appropriately chosen 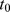, a node with a large out-component in 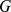 will also have a large one in 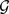, but for an inappropriately chosen 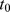 no or almost no other nodes can be reached. Vernon et al. [11] and Bajardi et al. [14] found similar results and explain them with the importance of connecting to the right node, i.e. a hub, at the right time.

To allow for a more complete view on the network, Figure 2B depicts 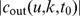 for all nodes 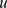. This is a top view of Figure 2A for all nodes of the network. The nodes are arranged along the vertical axis in a descending order from top to bottom according to their mean value 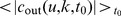. The dotted line marks the example node that is shown in panel A.

Most nodes exhibited vanishing out-components for almost all times of infection. This is indicated by the bright region in the lower half of Figure 2B. Only the top 30% of nodes possess a reasonably large out-component. Overall, it became clear that only a small fraction of nodes contributed to the risk of spreading in the network. This feature would be missed in a time-aggregated network study.

### Ranking of Nodes According to the Out-component

To allow for further investigation of the size of the out-component 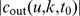, we focused on the effect of varying infectious periods 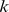. We averaged over the starting times 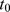 and thus determined the mean values 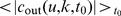. This limitation is justified by the likely unavailability of information on 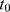 in every real-world surveillance scenario, where it is often hard to determine the precise time of primary infection. Hence, the exact value of 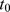 is inaccessible.

One should recall that the values of any risk-based measure as such are usually of minor importance for disease control. In most cases it is sufficient to identify the nodes that exhibit the highest values with respect to a particular measure. In fact, these will be the ones where interventions are most promising. In order to locate these nodes, it is sufficient to order the nodes for each 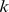 by the value of 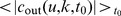 in a ranking 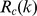.

**Figure 3:**
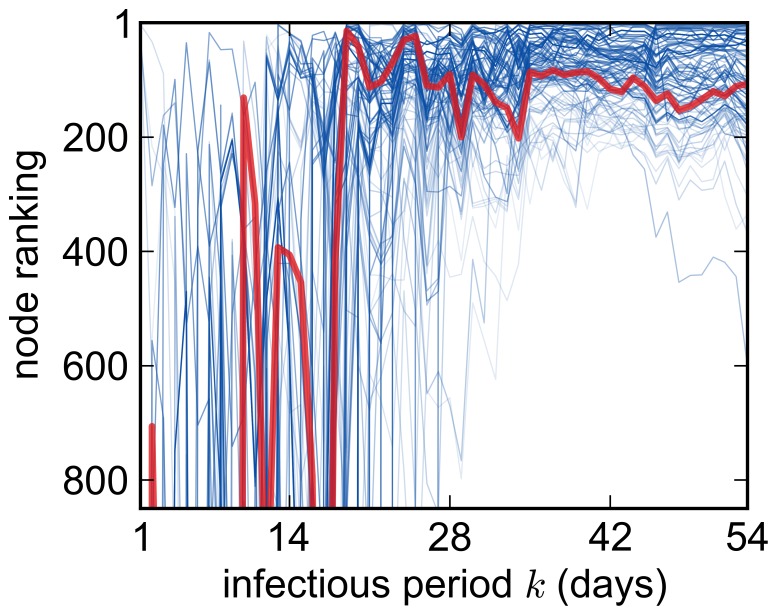
Ranking of nodes according to their mean out-component size 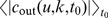. Each curve corresponds to one node. The top hundred nodes with the largest out-components are shown. Curves representing nodes with higher ranking are darker than those with lower rankings. For illustration purposes an arbitrarily chosen node is displayed in red.

Figure 3 presents the rankings 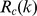 of the top 100 nodes. These nodes had on average the largest out-component, where the average is also calculated over all infectious periods 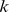, i.e. 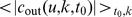. Each curve corresponds to one node and the red curve represents an arbitrarily chosen node highlighted for illustration purposes. For infectious periods 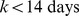, the ranking was very unstable and the average over both 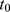 and 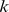 was not reliable. As 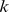 increased, however, it became more and more stable. In this regime, the rank of the top nodes did not change significantly. This means that for infectious periods that are long enough, the importance of explicitly considering a ranking for a given 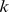 decreases.

### Robustness of Sampling Under Inaccurate Infectious Periods

For the purpose of disease control, it is desirable to know the set of nodes 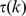 with the highest ranking according to a risk-based measure, where the number of top-ranked nodes, i.e. 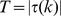 is predetermined by the given resources, e.g. the available number of vaccine doses. It is therefore crucial to investigate to what extend the composition of such a top sample depends on the infectious period 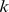, or more accurately, it is important to analyze the sensitivity of 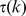 on an error 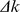. The value of 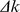 is given by the accuracy, in which the infectious period 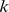 can be estimated. The sample 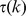 is determined by thresholding the ranking. Only nodes with a ranking above threshold will be included into the sample.

This sensitivity can be analyzed by investigating the intersection 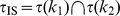 for any pair 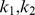. The ranking is independent of 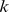, if 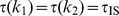 for every pair 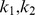. In general, however, this will not be the case, and the size of the intersection 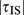 will be a function of 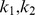. Additionally it will also depend on the sample size 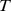.

The size of the intersection 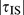 can be conveniently normalized by 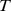, so that 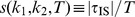 is the relative intersection. The measure 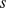 can be used to characterize the similarity of two rankings.

A further reduction of the dimensionality is possible by recalling disease control requirements. Since one is primarily interested in the sensitivity of 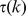 with respect to an error 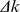, 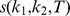 can be averaged over all pairs 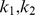 with 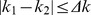 for a given sample size 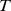. This yields a quantity 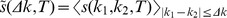 that corresponds to the robustness of a top sample with respect to inaccuracies in the infectious period.

Analogous averaging over all 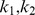 with 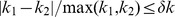 yields 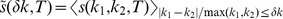 corresponding to the robustness of a top sample with respect to the relative error 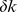.

**Figure 4:**
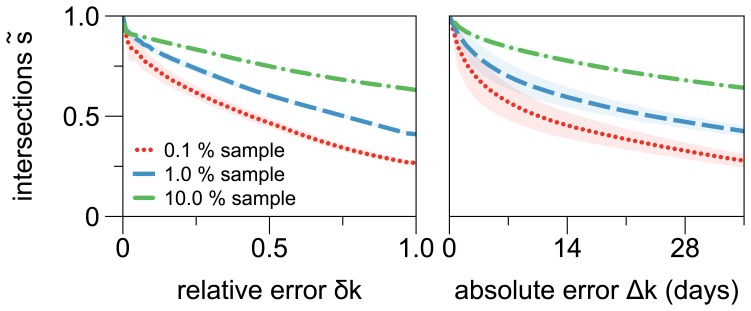
Robustness of samples based on the out-component size of nodes. Shown are the mean intersections 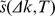 and 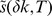 for three different sample sizes 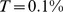 (red), 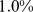 (blue), and 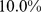 (green) of the network representing approximately 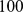, 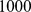, or 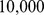 nodes, respectively. The sampling is calculated based on the mean largest out-component over all 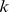 and 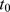 (see text for details). 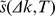 is based on averaging over all pairs 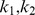 with 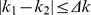 or 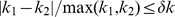 respectively. Confidence intervals are given by the shaded areas.

Figure 4 presents the relative intersections 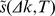 and 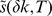 for three different sample sizes 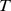, that is for 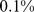, 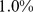, and 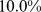 of all nodes. The 50% confidence interval is given by the shaded areas.

One can see that 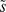 decreased with larger errors 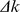. This holds for all three sample sizes. But also for very small samples in the order of 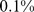 of the network, a robustness of 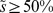 existed for errors of 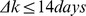. This means that, on average, half of the nodes in any such sample were identical and independent of the exact value of the infectious period used for evaluation. The definite number of identical nodes in such a sample could be analogously determined and yielded 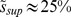 for 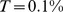 (not shown).

To account for the high variability in the ranking 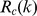 for small infectious periods (see Figure 3), one could consider sampling only for 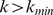 to include only the nodes with the highest overall risk in the sample. *Supporting Information S3 (Figure S5)* shows that this would further improve the robustness of a sample. However, no fundamental differences occur.

### Comparison of the Dynamic Out-component and Static Measures

**Figure 5:**
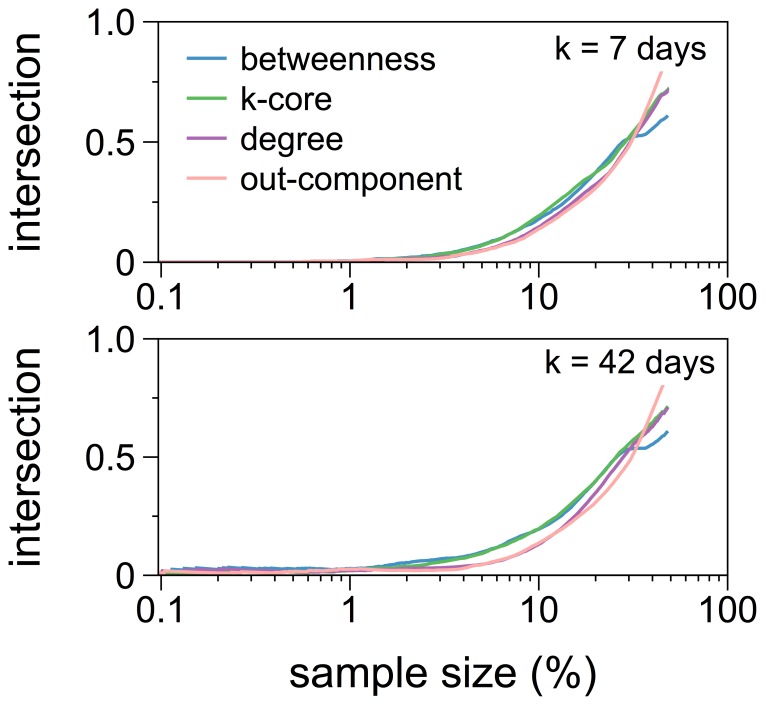
Comparison between the dynamic out-component and static measures. The relative size of the intersection of the top nodes is based on their value of the dynamic out-component and on static measures of centrality. In the upper panel the comparison for a fixed infectious period of 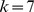 days is shown and in the lower one for a fixed infectious period of 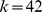 days.

Finally we compared our proposed measure to centrality measures on a static network representation. We followed the approach described in the previous section. Accordingly we determined the intersection of two top samples, i.e. the highest ranked nodes. One top sample is based on the dynamic out-component, the other on a selection of static centrality measures. In detail we compared 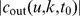 with the static out-component, the out-degree, betweenness and k-core centrality. In Figure 5 we show the respective intersections as a function of the sample size for two different infectious periods 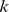. Only for samples bigger than 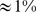 of the network, i.e. more than 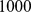 nodes the intersection took non-vanishing values. Hence central nodes in static network representations are likely to be different from those with a large temporal out-component.

## Conclusion and Discussion

We analyzed if time-independent determination of central nodes is possible even in a network with high temporal heterogeneity. The network under consideration is the German pig trade network. We investigated an epidemiological relevant centrality measure on this network with a topology that changes on the timescale of epidemic spreading. The spreading is described by a state-discrete SIR-like model. We focused on the out-component of a node as a measure of centrality for two reasons. First of all, an intuitive adaptability of the out-component exists in the time-aggregated case to finite infectious periods. Secondly, under the assumption of an infection probability 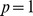 upon trading contact, the number of nodes in the out-component served as an upper bound to the size of an epidemic.

We found that the rapidly changing network topology, whose timescale is in the order of a typical infectious period, was reflected in the observed temporal heterogeneity of the out-component. We also demonstrated that the dynamic out-component only barely correlated with any static centrality measure. Therefore any static approximation should be used with caution. For the German pig trade network, however, a ranking based on the size of the out-component would be stable enough for disease control requirements.

Furthermore, the stable ranking allowed the sampling of nodes. We found such samples to be robust against variations in the length of the infectious period. For the German pig trade network, this enables the determination of disease-independent high-risk samples.

We emphasize that the results presented here are only valid for the specific network under consideration. Nevertheless, we expect similar results for other networks of animal trade, especially for pig trade networks due to the highly standardized and industrialized nature of these networks.

Our work contributes to improve surveillance and control of diseases, which propagate via trade of live animals. In the context of surveillance, one might argue that the in-component is a more suitable measure of centrality, but as shown in *Supporting Information S2 (Figures S2–S4)*, we find similar results for this case. We also briefly investigated SIS-like spreading, where reinfection of nodes is possible. Also here the results are similar.

This paper is based on three assumptions that are critical for the applicability of its results. First, the data used was collected during a disease-free period of the network. It is known, however, that the topology of animal trade networks changes significantly if a disease is detected [48]. Therefore the term infectious period is misleading and actually refers to what is called the high-risk period of a disease, that is, the time span between the primary infection in the network and the disease detection.

The second assumption is the homogeneity of the nodes. In reality, the nodes of an animal trade network exhibit different functionalities, e.g. breeders and slaughterhouses. This yields very different infection probabilities. This paper circumvents this problem by assuming an infection probability of 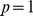 on contact. Therefore, our results can be seen as a worst case scenario.

Finally, the analysis of the robustness is based on averaging the size of the out-component over several days of primary infection. This approach is supported by the likely unavailability of any information on the exact day of a primary infection in the case of disease surveillance. However, if this information had been available, averaging might represent an unnecessary limitation (see *Supporting Information S3 (Figure S6)* for details).

An additional remark has to be made on the assumption that a transmission probability of 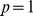 depicts the worst-case scenario. For an SIR-like spreading process on a temporal network it is possible that recovered nodes form a transmission barrier and thus preventing a disease from infecting a much larger portion of the network. Therefore a lower value of 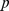 may also cause a much larger outbreak. This effect is discussed in detail in [38].

In conclusion, we showed that the notion of time-independent node centrality is critical in the context of temporal networks. However, stationary sampling of nodes remains still possible for the presented network.

Our findings can be applied in a more accurate risk assessment of a disease outbreak in the absence of counteractions. As a next step, the effect of vaccination protocols could also be taken into account as well as the implementation of a sophisticated surveillance system.

## Supporting Information

Supporting Information S1 **Representative Sample in Temporal Network Data (with Figure S1).**
(PDF)

Supporting Information S2 **Analysis of the In-Component (with Figures S2–S4).**
(PDF)

Supporting Information S3 **Additional Investigation of the Dynamic Out-Component (with Figures S5–S6).**
(PDF)
