# Supplementary material for: On the Robustness of In- and Out-Components in a Temporal Network
Source: PLoS One. 2013 Feb 6;8(2):e55223. doi: 10.1371/journal.pone.0055223 (PMC3566222; doi:10.1371/journal.pone.0055223)
Supplement: Supporting Information S1 — Representative Sample in Temporal Network Data (with Figure S1). (PDF) [file pone.0055223.s001.pdf]

S1 Representative Sample in Temporal Network Data

The HIT database records all trade movements for cattle since 2001 and for pigs since 2006. For data reaching far back and for those data, which are still recorded, the problem of determining a representative sample is ubiquitous. To determine the size of the sample we found two different approaches useful.

To investigate the periodicity of the German pig trade network we shift a week-long accumulation window across the data and count the number of active nodes and edges within the window that appear during the period. The result is a fingerprint of the one-week-periodicity of the network as shown in Figure S1 A. Nodes and edges display very similar characteristics. Besides the marked public holidays during Easter and Christmas, a dip during the summer months can be identified. Furthermore a general decline in the trade activity can be recognized, which is in agreement with an increasing centralization in the production process. This results in a smaller number of active nodes in the trade network.

An alternative approach is followed in Figure S1 B. To analyze the networks convergence, we use an accumulation window with increasing size. The orange and green dashed curves show the percentage of nodes and edges that appear since the first day of the considered period, respectively. Further understanding of the convergence can be achieved by looking at the accumulation rate as also shown in Figure S1 B as solid curves. The accumulation rate gives the relative increase of the numbers within one day.

It is clearly visible that after approximately half a year nodes and edges are added to the network with constant rates below 0.1%. This rate is governed by the long-time growth of the network.

Considering both analyses we can assess that a period of one year is sufficient to cover all characteristic properties of the short- and medium-scale temporal developments of the German pig trade network. Long-scale developments are of minor importance from an epidemiological point of view, since their scales outnumber any plausible infectious period.

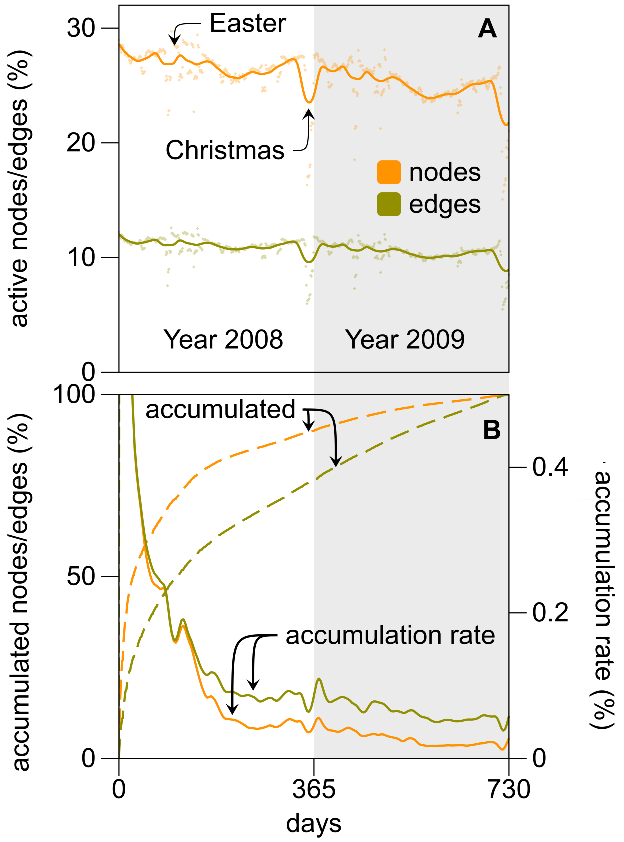

Figure S1. Periodicity and convergence in the German pig trade network.
Panel A: Percentage of nodes (orange) and edges (green) that appear during a week-long sliding average. The periodicity has been investigated by a shifted accumulation window. To compensate trivial weekly fluctuations a window of seven days width has been used. In such a window all nodes and edges in the dataset for times $t_d \leq t < t_{d+7}$ are accumulated into a time-aggregated network. The increment d hereby scans the complete dataset $0 \leq d \leq d_{\max}$, where $d = 0$ is equal to 01/01/2008 and $d = d_{\max}$ is equal to 12/31/2009.
The line represents a local regression of the original data, which are shown as points.
Panel B: Number of nodes (orange) and edges (green) in the time-aggregated network of increasing length and the accumulation rate, i.e. the number of nodes and edges added each day. The analysis of the convergence is utilizing an accumulation window with increasing size. In this window all nodes and edges in the dataset for times $t_0 \leq t < t_d$ are aggregated into a time-aggregated network. This means we consider first the network of just the first day (01/01/2008), then the time-aggregated network of the first and the second day and so forth.
The solid curve displays a local regression of the original data which itself is not shown to maintain clarity.
