# Supplementary material for: On the Robustness of In- and Out-Components in a Temporal Network
Source: PLoS One. 2013 Feb 6;8(2):e55223. doi: 10.1371/journal.pone.0055223 (PMC3566222; doi:10.1371/journal.pone.0055223)
Supplement: Supporting Information S2 — Analysis of the In-Component (with Figures S2–S4). (PDF) [file pone.0055223.s002.pdf]

S1 Analysis of the In-Component

Temporal heterogeneity of the in-component

In Figure S2 A the distribution of the size of the in-component $|c_{\text{in}}(v,k,t_0)|$ for an exemplary node u and a fixed infectious period $k = 24$ days is shown. In contrast to $|c_{\text{out}}(u,k,t_0)|$ no line-like structure is found for the in-components. Instead $|c_{\text{out}}(u,k,t_0)|$ takes an oscillatory form. This is true for all nodes as the washed-out appearance of Figure S2 B illustrates. The later is a top view of Figure S2 A for all nodes of the network. The nodes are arranged along the vertical axis in a descending order from top to bottom according to their mean value $\langle|c_{\text{in}}(u,k,t_0)|\rangle_{t_0}$. The dotted line marks the example node, that is shown in panel A.

Another major difference to $c_{\text{out}}(u,k,t_0)$ is the concentration of centrality on even less nodes as in the case of the out-component. The reason is the asymmetric nature of the network, where comparatively few slaughterhouse nodes ($< 1\%$) act as drains for the whole network. These are in the range of almost every infection path in the network. Therefore eventually every primary infection will end in one of these nodes giving them a very high value of centrality relative to the rest of the network.

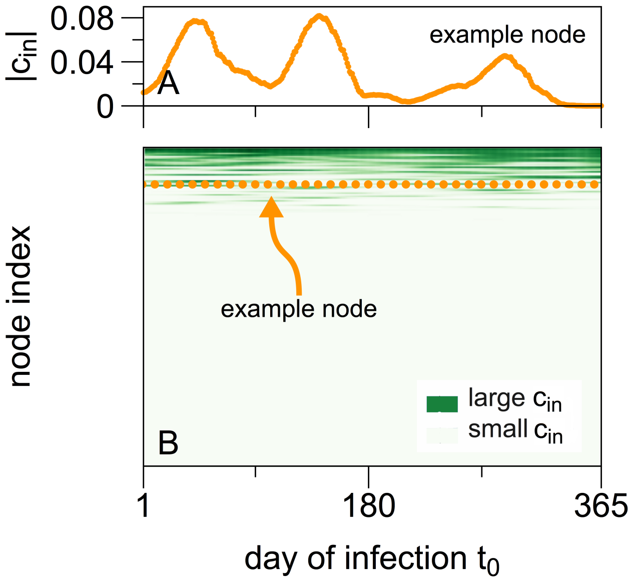

Figure S2. Distribution of $|c_{\text{in}}(u,k,t_0)|$ for an exemplary infectious period of $k = 24$ days.
Panel A shows the size of the in-component for an exemplary node u as a function of t_0. Panel B shows the distribution for all nodes, i.e. the top view of panel A for all nodes of the network. Each horizontal line represents one node, the example node chosen for panel A is indicated by the dotted orange line. For the sake of clarity, only every 100th node is plotted. Nodes are arranged according to their averaged value of $\langle|c_{\text{in}}(u,k,t_0)|\rangle_{t_0}$ over all t_0 from top to bottom, i.e. the node with the largest averaged in-component is displayed as the top line of the panel.

Ranking of nodes according to the in-component

Figure S3 presents the rankings $R_c(k)$ of the top nodes according to their mean in-component size $\langle|c_{\text{in}}(u,k,t_0)|\rangle_{t_0}$. Every line corresponds to one node, where only the top hundred nodes are shown. The red line represents an arbitrarily chosen node highlighted for illustration purposes. Similar to the case of the out-component discussed in the main text, the ranking for the in-component is very unstable for all nodes for small infectious periods $k \gtrsim 21$ days. As k increases it becomes more and more stable.

A remarkable difference to the ranking according to the out-component is again the more centralized, i.e., more stable appearance.

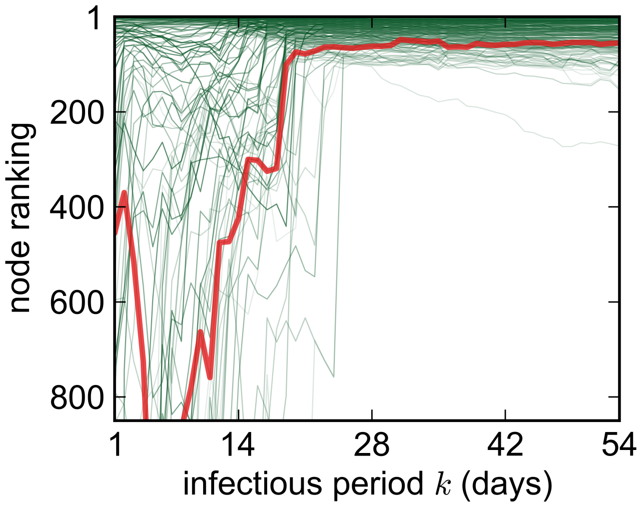

Figure S3. Ranking of nodes according to their mean out-component size.
Every line corresponds to one node. The hundred highest ranked nodes are shown based on the average $\langle|c_{\text{in}}(u,k,t_0)|\rangle_{t_0}$. Curves representing nodes with a higher ranking are darker than those with lower rankings. For illustration purposes, an arbitrarily chosen node is displayed in red.

Robustness of sampling under inaccurate infectious periods

Figure S4 presents the relative intersections $\tilde{s}(\Delta k)$ and $\tilde{s}(\delta k, T)$ for three different sample sizes T. A detailed description is given in the results section. The robustness of samples based on the in-component is much higher compared to samples based on the out-component. This is again related to the centralized drain structure of the investigated network.

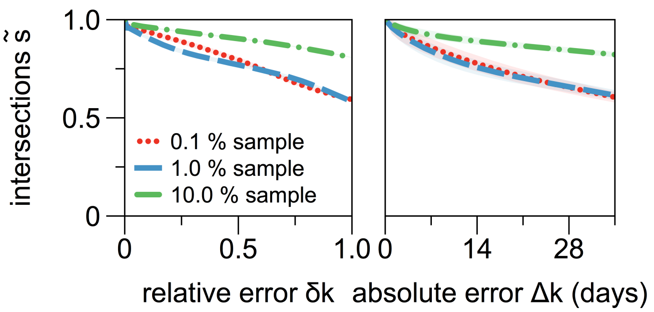

Figure S4. Robustness of samples based on the out-component size of nodes.
Shown are the mean intersections $\tilde{s}(\Delta k, T)$ and $\tilde{s}(\delta k, T)$ for three different sample sizes $T = 0.1\%$ (red), 1.0% (blue), and 10.0% (green) of the network representing approximately 100, 1000, or 10,000 nodes, respectively. The sampling is calculated based on the mean largest in-component over all k and t_0 (see text for details). $\tilde{s}(\Delta k, T)$ is based on averaging over all pairs k_1, k_2 with $|k_1 - k_2| \leq \Delta k$ or $|k_1 - k_2| / \max(k_1, k_2) \leq \delta k$ respectively. The confidence interval is given by the shaded areas.
