# Supplementary material for: On the Robustness of In- and Out-Components in a Temporal Network
Source: PLoS One. 2013 Feb 6;8(2):e55223. doi: 10.1371/journal.pone.0055223 (PMC3566222; doi:10.1371/journal.pone.0055223)
Supplement: Supporting Information S3 — Additional Investigation of the Dynamic Out-Component (with Figures S5–S6). (PDF) [file pone.0055223.s003.pdf]

S1 Additional Investigation of the Dynamic Out-Component

Intersection of samples for $k \geq k_{min}$

To take into account the high instability in the ranking of nodes for smal infectious periods k we consider creating top samples with a lower bound k_{min}. To do so we repeat the calculation of the relative intersection $\tilde{s}$ but average only over $|k_1 - k_2| \leq \Delta k$ with $k \geq k_{min}$. Figure S6 shows the result for a sample size of 0.1% of the network. Any k_{min} will increase the intersection of the top samples.

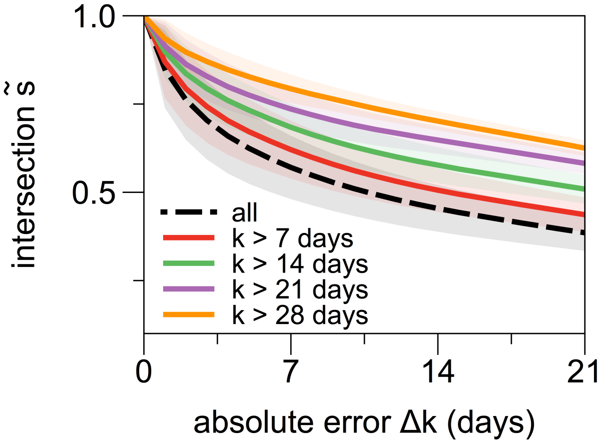

Figure S5. Intersection of samples for $k \geq k_{min}$.
The mean relative size of the intersection of top samples for a limited range of infectious periods with $k \geq k_{min}$. The shaded area respresents the confidence interval of each curve respectively.

Intersection of samples based on different days of primary infection t_0

Since the dynamic out-component also depends on the day of the primary infection, it also useful to investigate its dependence on t_0 for a fixed infectious period $k = \text{const}$. The analysis follows the steps of the Results section but instead of averaging over all k based on a ranking, which itself is already averaged over different t_0, we calculate the relative intersections $\tilde{s}$ for different days of of primary infection t_0 directly. The infectious period k is fixed. We obtain Figure S6. The relative size of the intersection significantly drops if two days of primary infection differ by more than a week.

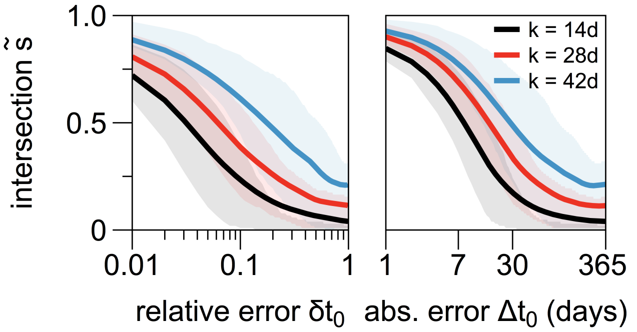

Figure S6. Intersection of samples based on different days of primary infection t_0.
The mean relative size of the intersection for the top 0.1% of all nodes for three different infectious periods k. The confidence interval of the mean is given by the shaded areas. The left panel shows the intersection of samples as a function of relative error $|t_{0,1} - t_{0,2}| / \max(t_{0,1}, t_{0,2}) \leq \delta t_0$ and the right panel as a function of the absolute error $|t_{0,1} - t_{0,2}| \leq \Delta t_0$
